# Impact of body mass index on outcomes after radical cystectomy: A retrospective Australian cohort study

**DOI:** 10.1002/bco2.70238

**Published:** 2026-07-08

**Authors:** Samiha Arulshankar, Xinyi Wei, Charis Ting, Tran Ngoc An Huynh, Yashvrdhan Khanna, Kylie Lim, Munad Khan, James Huang, Nieroshan Rajarubendra, Kevin Chu, Matthew Harper, Scott Donnellan, Weranja Ranasinghe

**Affiliations:** ^1^ Department of Urology Monash Health Victoria Australia; ^2^ Faculty of Medicine, Nursing and Health Science Monash University Melbourne Australia

**Keywords:** bladder cancer, body mass index, obesity paradox, perioperative outcomes, radical cystectomy, survival

## Abstract

**Objectives:**

This study aimed to assess the association between body mass index and perioperative outcomes and survival after radical cystectomy for bladder cancer in an Australian cohort.

**Subjects/patients and methods:**

We conducted a retrospective single‐centre study of patients undergoing radical cystectomy between 2008 and 2021. Preoperative body mass index (BMI) was analysed categorically and continuously. Outcomes included overall survival (OS), Clavien–Dindo grade ≥2 complications and length of hospital stay. Survival was analysed using Kaplan–Meier methods and multivariable Cox regression adjusting for age, sex, tumour stage, nodal status and chemotherapy. Restricted cubic splines were used to explore non‐linear associations.

**Results:**

The cohort comprised 135 patients (median age 70 years; median BMI 27.2 kg/m^2^). Overweight patients had superior OS compared with normal‐weight and obese patients (adjusted hazard ratio [HR] 0.42, 95% CI 0.24–0.76). Obesity was associated with higher complication rates and longer hospital stay (*p* < 0.05). BMI analysed as a continuous variable was not independently associated with OS. Spline modelling demonstrated no significant non‐linear association, although the lowest estimated risk occurred in the overweight range.

**Conclusion:**

Overweight BMI was associated with improved survival following radical cystectomy, while obesity was linked to greater perioperative morbidity. These findings are consistent with the obesity paradox and underscore the limitations of BMI alone for perioperative risk stratification.

## 1 INTRODUCTION

Radical cystectomy (RC) is the standard surgical treatment for muscle‐invasive and high‐risk bladder cancer but remains a complex urological procedure associated with substantial morbidity and mortality.^1^, ^2^ As global obesity trends continue to rise, understanding its implications for surgical outcomes and long‐term survival is increasingly relevant for risk stratification.

The impact of BMI on RC outcomes has been debated extensively. Some studies report that obesity predisposes patients to higher surgical complexity, increased perioperative complications and worse oncological outcomes.^3^, ^4^ Conversely, others describe an ‘obesity paradox’, wherein overweight and mildly obese patients demonstrate improved survival compared to their normal‐weight counterparts.^5^, ^6^, ^7^ These conflicting findings complicate preoperative counselling and risk stratification in patients undergoing RC and may reflect biological differences in tumour behaviour, alterations in inflammatory and metabolic pathways or methodological biases such as reverse causality and selection effects.^8^, ^9^, ^10^

Despite international data, there remains a lack of contemporary Australian research investigating the relationship between BMI and RC outcomes. Our study aims to explore this association in an Australian cohort, focusing on overall survival, perioperative morbidity and length of hospital stay, while accounting for potential confounders and assessing the possibility of non‐linear relationships between BMI and outcomes.

Although we acknowledge the limitations of BMI as a sole measure of adiposity, it was selected as the primary exposure variable due to its universal availability in retrospective datasets, enabling consistent classification across the entire study period. This study also provides contemporary Australian data in a domain where published evidence from this geographic and healthcare context remains limited.^11^

## 2 SUBJECTS/PATIENTS AND METHODS

**FIGURE 1:**
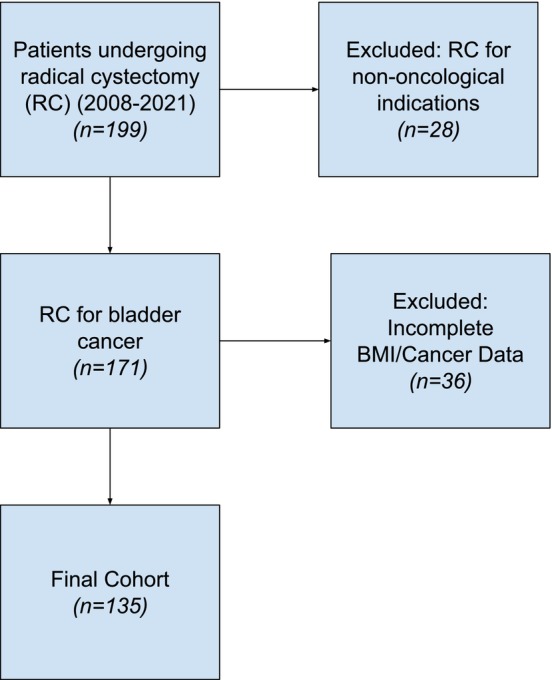
Flowchart of patient selection for the study cohort.

We performed a retrospective review of all patients who underwent radical cystectomy at a single tertiary centre in Australia between January 2008 and December 2021. Of 199 patients initially identified, 28 were excluded due to non‐oncological indications, and 36 were excluded for incomplete BMI or cancer data, leaving 135 patients in the final analysis (Figure 1).

Data collected included age, sex, preoperative BMI, Charlson Comorbidity Index (CCI), tumour stage, nodal status, use of neoadjuvant chemotherapy (NAC), type of urinary diversion, postoperative complications (graded by Clavien–Dindo), length of stay and overall survival (OS). BMI was recorded at preoperative assessment and classified as normal (18.5–24.9 kg/m^2^), overweight (25.0–29.9 kg/m^2^) or obese (≥30 kg/m^2^) per WHO criteria.

All radical cystectomies were performed via an open (laparotomy) approach. Surgical approach, urinary diversion type (ileal conduit or orthotopic neobladder), and history of prior abdominal surgery were recorded and analysed as potential confounders of perioperative outcomes. Pelvic radiotherapy history was not systematically captured in this dataset and could not be included in the analysis; this represents a limitation acknowledged below.

Statistical analyses were conducted using R software. Group comparisons employed chi‐square, Fisher's exact or ANOVA tests. Survival analyses utilised Kaplan–Meier curves, log‐rank tests and Cox proportional hazards models adjusting for age, CCI, stage and NAC. BMI was examined both categorically and as a continuous variable, including multivariable restricted cubic spline modelling. Significance was set at *p* < 0.05.

This study was approved by the local institutional research ethics board (Ethic Committee Name: Monash Health Human Research Ethics Committee; Approval Code: RES‐23‐0000‐581Q).

## 3 RESULTS

### 3.1 Cohort characteristics

A total of 135 patients undergoing RC for bladder cancer (BC) between 2008 and 2021 were included in the final analysis. The median age was 70 years (range 45–85). The overall median BMI was 27.2 kg/m^2^. Patients were distributed as follows: 28% (*n* = 38) with normal BMI, 40% (*n* = 54) overweight and 32% (*n* = 43) obese. Baseline comorbidities, measured by the CCI, were similar across groups, with mean scores of 5.3 in normal BMI, 5.6 in overweight and 5.5 in obese patients (Table 1).

**TABLE 1:** Baseline clinical, tumour and perioperative characteristics by BMI category.

Characteristic*	BMI ≤ 24.9	25 ≤ BMI ≤ 29.9	BMI ≥ 30	*p*‐value
Patients in each BMI category	38 (28.1%)	54 (40.0%)	43 (31.9%)	‐
Mean age, years	69.4 ± 12.5	73.4 ± 8.1	71.9 ± 10.3	0.58
Gender (male/female)	31 (81.6%)/7 (18.4%)	47 (87.0%)/7 (13.0%)	31 (72.1%) /12 (27.9%)	0.7
**Surgical details**				
Surgical approach, *n* (%)				
Open (laparotomy)	38 (100%)	54 (100%)	43 (100%)	—
Urinary diversion, *n* (%)				
Ileal conduit	35 (92.1%)	48 (88.9%)	40 (93.0%)	0.64
Neobladder	1 (2.6%)	3 (5.6%)	1 (2.3%)	
Not recorded	2	3	2	
Prior abdominal surgery, *n* (%)				
Yes	11 (28.9%)	21 (39.6%)	12 (27.9%)	0.40
No	27 (71.1%)	32 (60.4%)	31 (72.1%)	
Not recorded	0	1	0	
**TURBT stage**				
<T2	3 (8.3%)	10 (19.2%)	10 (23.8%)	0.19
≥T2	33 (91.7%)	42 (80.8%)	32 (76.2%)	
**Pathological tumour stage**				
T0	2 (5.3%)	11 (20.4%)	5 (11.6%)	0.11
Tis/Ta/T1	10 (26.3%)	14 (25.9%)	9 (20.9%)	0.81
T2	6 (15.8%)	13 (24.1%)	8 (18.6%)	0.59
T3	16 (42.1%)	12 (22.2%)	14 (32.6%)	0.12
T4	3 (7.9%)	2 (3.7%)	7 (16.3%)	0.09
**Pathological node status**				
Node negative	31 (81.6%)	44 (81.5%)	31 (72.1%)	0.46
Node positive	7 (18.4%)	10 (18.5%)	12 (27.9%)	0.46
**Histology**				
Urothelial	27 (71.1%)	40 (74.1%)	36 (84.7%)	0.36
Variant	6 (15.8%)	4 (7.4%)	7 (16.3%)	0.33
**Chemotherapy**				
Neoadjuvant chemotherapy (%)	1 (2.6%)	8 (14.8%)	4 (9.3%)	0.15
Adjuvant chemotherapy (%)	9 (23.7%)	10 (18.5%)	16 (37.2%)	0.11
**Perioperative outcomes**				
Mean length of hospital stay, days	9.29 ± 3.5	9.45 ± 4.4	14.4 ± 13.15	<0.05
Complications (Clavien–Dindo ≥2)	8 (21.0%)	8 (14.8%)	16 (37.2%)	<0.05
**Complications by type**				
GIT complications, *n* (%)				0.94
Paralytic ileus	9 (23.7%)	16 (29.6%)	12 (27.9%)	0.82
Anastomotic leak	1 (2.6%)	0 (0.0%)	0 (0.0%)	0.28
Infection, *n* (%)				0.24
Urinary tract infection	3 (7.9%)	1 (1.9%)	1 (2.3%)	0.27
Wound/surgical site infection	1 (2.6%)	1 (1.9%)	3 (7.0%)	0.38
Sepsis/bacteraemia	2 (5.3%)	2 (3.7%)	2 (4.7%)	0.94
Pneumonia	0 (0.0%)	0 (0.0%)	2 (4.7%)	0.11
Cardiopulmonary, *n* (%)				0.33
AF/arrhythmia	5 (13.2%)	3 (5.6%)	6 (14.0%)	0.32
Respiratory failure	1 (2.6%)	1 (1.9%)	1 (2.3%)	0.97
Urological/Surgical, *n* (%)				0.38
Urine leak	3 (7.9%)	1 (1.9%)	2 (4.7%)	0.38
Metabolic, *n* (%)				<0.001
Acute kidney injury	0 (0.0%)	2 (3.7%)	9 (20.9%)	<0.001
Electrolyte disturbance	0 (0.0%)	5 (9.3%)	5 (11.6%)	0.11
ICU admission, *n* (%)	4 (10.5%)	6 (11.1%)	9 (20.9%)	0.29
Blood transfusion, *n* (%)	8 (21.1%)	6 (11.1%)	12 (27.9%)	0.11
Reoperation for complication, *n* (%)	1 (2.6%)	0 (0.0%)	0 (0.0%)	0.28

*Note: p*‐values derived from chi‐square or Fisher's exact test (categorical variables) and one‐way ANOVA (continuous variables). For complication categories, the *p*‐value on the category heading row reflects any complication within that category; subitem *p*‐values reflect that specific complication.

Abbreviations: AF, atrial fibrillation; AKI, acute kidney injury; BMI, body mass index; GIT, gastrointestinal; ICU, intensive care unit.

* Data presented as mean ± standard deviation or number (%).

### 3.2 Tumour characteristics and treatment

Tumour characteristics were generally comparable across BMI groups. Pathological tumour staging showed no significant differences between groups, with similar distributions of T0–T4 disease. The rates of node‐positive status were 27.9% in the obese, 18.5% in the overweight and 18.4% in the normal BMI patients (*p* = 0.46). NAC use was 14.8% in the overweight group, 2.6% in the normal BMI group and 9.3% in the obese group (*p* = 0.15). The rates of tumour upstaging were 53.9% in the obese, 35.0% in the overweight, and 47.4% in the normal BMI patients (*p* = 0.44). Logistic regression demonstrated a trend towards lower odds of upstaging in overweight patients compared with obese patients (OR 0.46), although this difference did not reach statistical significance (*p* = 0.094).

The overall low rate of NAC use (range 2.6%–14.8% across groups) reflects broader patterns of underutilisation documented in Australian practice.^11^, ^12^ As previously reported from our institution, barriers include patient comorbidity precluding cisplatin eligibility, patient preference and logistical challenges of accessing systemic chemotherapy at a semirural tertiary centre.^11^ The high rate of pathological upstaging is consistent with literature demonstrating that clinical staging underestimates pathological stage, reflecting the well‐recognised limitations of preoperative clinical staging in muscle‐invasive bladder cancer.^13^, ^14^, ^15^

Adjuvant chemotherapy was used infrequently and without significant variation between groups (Table 1). Where administered, indications included pathological stage pT3 or pT4a disease, lymph node‐positive disease (pN+) and positive surgical margins in patients who had not received NAC, consistent with contemporary guideline recommendations.

Histological subtype was predominantly urothelial carcinoma across all BMI groups, with variant histology seen in approximately 25% of cases without significant differences by BMI category.

### 3.3 Perioperative outcomes

Obese patients experienced significantly longer hospitalisations (mean 14.6 ± 3.8 vs. 9.2 ± 2.5 days for normal‐weight and 9.3 ± 4.5 days for overweight, *p* < 0.05) and higher rates of Clavien–Dindo grade ≥2 complications (37.2% vs. 21.0% and 14.8%, *p* < 0.05) as summarised in Table 1.

The most common GIT complication was paralytic ileus across all groups. Infectious complications included urinary tract infection, wound infection and sepsis, with pneumonia observed only in the obese group (4.7%). Cardiopulmonary complications were predominantly AF/arrhythmia, which occurred at broadly similar rates across all groups (Normal: 13.2%, Overweight: 5.6% and Obese: 14.0%). Metabolic complications showed a marked BMI‐dependent gradient: acute kidney injury (AKI) occurred in 0%, 3.7% and 20.9% of normal, overweight and obese patients, respectively, consistent with published evidence identifying obesity as an independent preoperative risk factor for AKI following RC.^16^, ^17^ Electrolyte disturbance requiring intravenous correction was recorded in 0%, 9.3% and 11.6% of patients across the three groups. ICU admission was required in 10.5%, 11.1% and 20.9% of patients, and blood transfusion in 21.1%, 11.1% and 27.9% of the normal, overweight and obese groups, respectively. Reoperation for a complication was rare, occurring in one normal‐weight patient only.

### 3.4 Survival outcomes

**FIGURE 2:**
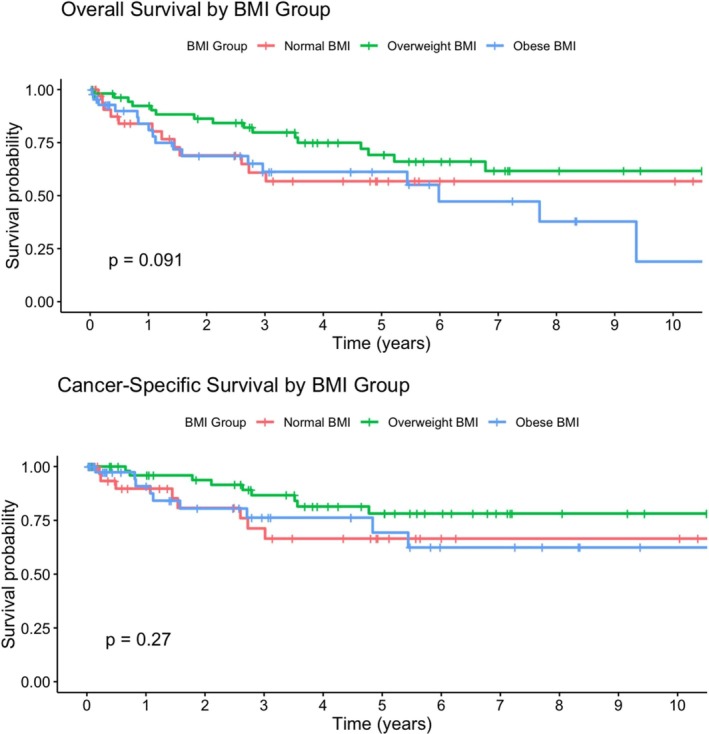
Kaplan–Meier overall survival and cancer‐specific survival curves stratified by BMI category.

Kaplan–Meier analysis demonstrated significant differences in overall survival (OS) among BMI groups (log‐rank *p* = 0.091) (Figure 2), whereas cancer‐specific survival (CSS) was not significant. Overweight patients had superior OS compared to both normal‐weight and obese patients.

On univariable Cox regression, overweight BMI was associated with improved overall survival, with a hazard ratio (HR) of 0.42 (95% CI 0.24–0.76), while normal BMI (HR 1.73, 95% CI 0.87–3.45) and obese BMI (HR 1.85, 95% CI 0.95–3.60) (Table 2).

**TABLE 2:** Univariable and multivariable cox regression hazard ratios for overall survival.

Variable	Univariable HR (95% CI)	Multivariable HR (95% CI)
**BMI category**		
Normal BMI	1.73 (0.87–3.45)	0.75 (0.33–1.68)
**Overweight BMI**	**0.42 (0.24–0.76)**	**0.44 (0.20–0.96)**
Obese BMI	1.85 (0.95–3.60)	Reference
Tumour stage	—	1.32 (0.94–1.86)
Nodal status	—	1.80 (0.77–4.10)
Neoadjuvant chemotherapy	—	1.45 (0.38–4.09)
Adjuvant chemotherapy	—	1.45 (0.62–3.33)
Gender	—	1.10 (0.52–2.20)
Age	—	1.01 (0.97–1.04)

Multivariable analysis confirmed this survival advantage for overweight patients, demonstrating an HR of 0.44 (95% CI 0.20–0.96) after adjusting for tumour stage, nodal status, chemotherapy, gender and age (Table 2). Neither normal nor obese BMI reached statistical significance in multivariable modelling.

**FIGURE 3:**
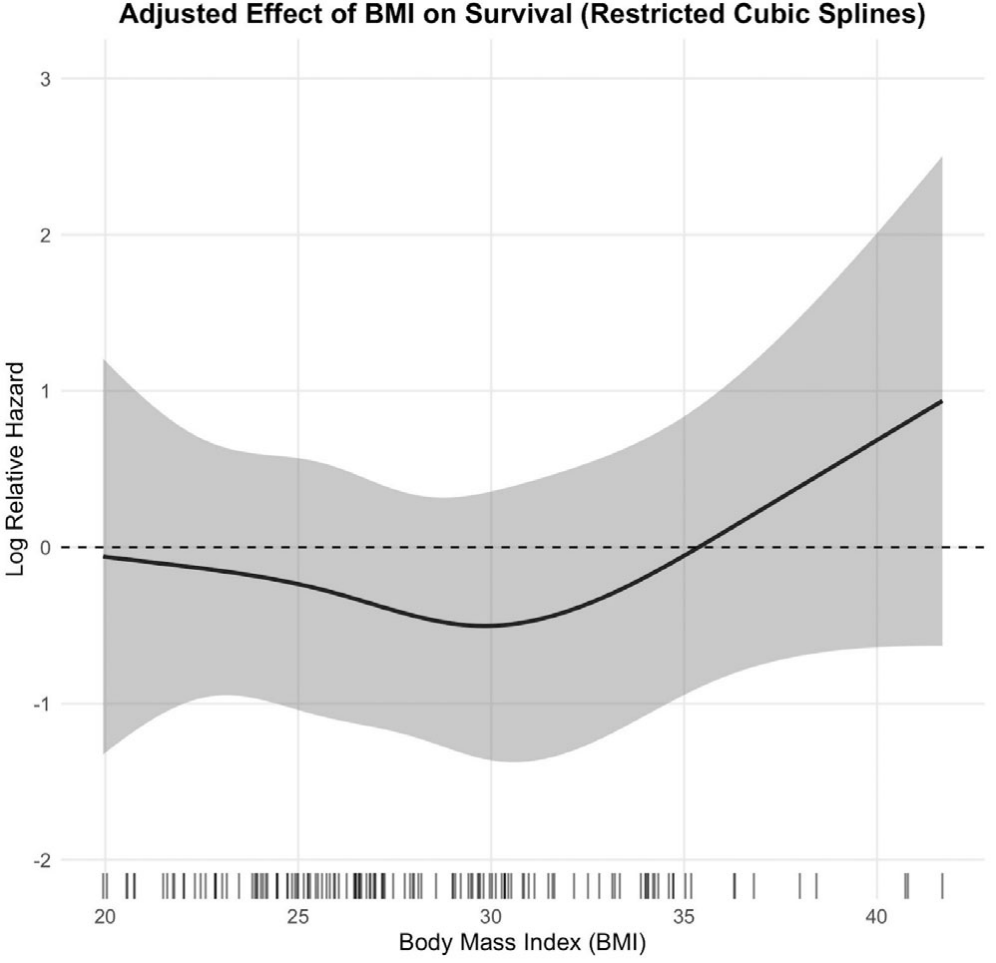
Restricted cubic spline model showing the adjusted relationship between BMI and hazard of death.

When BMI was analysed as a continuous variable, it was not an independent predictor of OS on either univariable (HR 1.01, 95% CI 0.93–1.08) or multivariable analysis (HR 1.04, 95% CI 0.96–1.11) (Table 3). Adjusted Cox proportional hazards modelling using restricted cubic splines (BMI knots at 5th, 35th, 65th and 95th percentiles) demonstrated no significant non‐linear association between BMI and overall survival (*p* = 0.40) with the overall model showing a moderate fit (likelihood ratio *χ* ^2^ = 31.54, df = 12, *p* = 0.002). However, hazard ratios relative to BMI 23 indicated a potential *U*‐shaped pattern, with the lowest risk at BMI 29.83 (HR = 0.70, 95% CI: 0.31–1.59) and elevated risks at extremes (e.g., BMI 20: HR = 1.09, 95% CI: 0.47–2.57; BMI 40: HR = 2.31, 95% CI: 0.68–7.86), though all confidence intervals crossed zero (Figure 3). A linear model confirmed no significant association (HR per unit BMI = 1.02, 95% CI: 0.95–1.10, *p* = 0.59).

**TABLE 3:** Cox regression hazard ratios treating BMI as a continuous variable.

Variable	Univariable HR (95% CI)	Multivariable HR (95% CI)
Body mass index (per kg/m^2^)	1.01 (0.93–1.08)	1.04 (0.96–1.11)
Tumour stage	—	1.37 (0.97–1.92)
Nodal status	—	1.63 (0.73–3.56)
Neoadjuvant chemotherapy	—	1.25 (0.34–3.57)
Adjuvant chemotherapy	—	1.57 (0.69–3.52)
Gender	—	1.07 (0.50–2.14)
Age	—	1.01 (0.98–1.04)

## 4 DISCUSSION

Our findings indicate that overweight patients undergoing RC for BC exhibit significantly better overall survival compared to those with normal or obese BMI. This observation aligns with reports of the obesity paradox, a phenomenon previously documented in several BC studies. Specifically, studies have shown either improved survival or favourable short‐term outcomes in overweight or obese patients, supporting the existence of this paradox.^18^, ^19^, ^20^, ^21^, ^22^, ^23^ Notably, Arthuso et al. demonstrated that this survival advantage may be short lived, with overweight and obese patients showing improved overall survival within the first 5 years following RC, but poorer survival beyond 5 years and therefore not captured in studies with short follow‐up.^18^ Nonetheless, the evidence is mixed, as other studies have reported no significant survival advantage or even worse oncological outcomes associated with higher BMI, effectively refuting the obesity paradox in BC.^4^, ^24^, ^25^, ^26^ These conflicting results highlight the complex and multifactorial relationship between body composition, tumour biology, perioperative risk and long‐term survival in patients undergoing RC.

The differential effect of BMI on OS but not CSS warrants interpretation. We hypothesise several explanations. First, overweight patients may have greater physiological reserve, affording resilience against noncancer causes of death, such as cardiovascular disease, frailty and sepsis, which are relevant competing causes of mortality in this predominantly elderly cohort. CSS, by definition, excludes noncancer deaths and therefore may not fully capture this effect. Second, the CSS analysis may be underpowered given fewer cancer‐specific death events. Third, residual confounding by comorbidity burden or performance status may have differentially influenced OS and CSS estimates.

One hypothesis is that overweight patients may possess greater physiological reserves to tolerate the metabolic stress of major surgery and systemic therapy. Emerging evidence suggests that obesity may influence tumour biology, as shown in renal cell carcinoma, where obesity has been linked to lower expression of fatty acid synthase (FASN), a marker of tumour aggressiveness.^9^ Whether similar molecular patterns exist in BC remains uncertain and warrants further research.

However, several methodological factors must be considered. BMI is a crude metric that does not distinguish between adiposity and lean muscle mass. Sarcopenia, the loss of skeletal muscle, has been independently associated with higher perioperative morbidity and poorer survival following RC. For example, Psutka et al. demonstrated that skeletal muscle depletion, rather than obesity alone, was a stronger predictor of adverse outcomes after RC.^21^ Thus, patients with similar BMI may have vastly different body compositions, leading to potential misclassification. Additionally, BMI was recorded only once preoperatively in our study and does not account for weight changes due to cancer cachexia, introducing possible reverse causation where lower BMI may reflect advanced disease rather than inherently better health status.

The relationship between obesity and systemic therapy outcomes remains complex. Some studies suggest that obesity may impair chemotherapy efficacy due to altered pharmacokinetics and reduced dose intensity.^22^ Conversely, higher BMI has been associated with improved responses to immune checkpoint inhibitors in several malignancies.^6^, ^23^ This may relate to chronic inflammation in obesity creating a tumour microenvironment that is more susceptible to immunotherapy.^23^ Recent findings also highlight the relationship between BMI, treatment receipt and health‐related quality of life among older BC patients, further complicating decision‐making in this patient group.^19^ As immunotherapy becomes increasingly integrated into BC management, it is important to establish whether the observed survival advantage in overweight patients persists in this evolving therapeutic landscape.

There was some variation in tumour upstaging across BMI groups which has had limited exploration in the literature thus far. Albeit not reaching statistical significance, 53.9% of obese BMI patients demonstrated pathological tumour upstaging post‐cystectomy compared to 35.0% of overweight BMI patients (*χ* ^2^ = 3.74, df = 4, *p* = 0.44). Logistic regression supported this trend, showing that overweight patients had lower odds of being upstaged compared to obese patients (OR = 0.46), although this did not reach statistical significance (*p* = 0.094). These findings raise the possibility that tumour biology or staging accuracy may vary by BMI contributing to the differing survival profiles of patients; however, further exploration with larger datasets would be required to validate these observations.

Our study demonstrated significantly higher rates of perioperative complications and longer hospital stays among obese patients. The lower perioperative complication rate and shorter length of stay observed in overweight patients may partly explain why this group experiences superior overall survival. At the granular level, metabolic complications, particularly AKI, showed a marked BMI‐dependent gradient (0%, 3.7% and 20.9% across normal, overweight and obese groups), consistent with data demonstrating obesity as an independent risk factor for AKI after RC.^16^, ^17^ Similarly, cardiopulmonary complications, predominantly AF/arrhythmia, occurred at broadly similar rates in normal (13.2%) and obese (14.0%) patients but were lowest in the overweight group (5.6%), which may further contribute to the differential survival advantage observed in this group. This finding is consistent with the established association between obesity and AF risk.^27^ Blood transfusion requirements (21.1%, 11.1% and 27.9%) and ICU admission rates (10.5%, 11.1% and 20.9%) also trended higher with increasing BMI, consistent with the overall perioperative risk profile in obese patients.^28^

These findings have significant implications for the healthcare system as obesity rates continue to rise, increasing the proportion of patients at higher perioperative risk.^29^ Longer hospital stays and higher complication rates in obese patients may further strain public hospital resources and contribute to rising healthcare costs. While obesity cannot be rapidly reversed before surgery, perioperative prehabilitation including exercise, nutritional support and psychological care has shown promise in improving surgical fitness and reducing complications.^26^ Targeted prehabilitation for obese patients, including nephroprotective perioperative strategies given the elevated AKI risk, could reduce complications and hospital stays, potentially improving both outcomes and resource utilisation.

## 5 LIMITATIONS

This study has several limitations typical of retrospective analyses. The modest sample size reduces statistical power, especially for subgroup and spline analyses. BMI, although widely used, does not distinguish between adipose and muscle tissue, and we lacked CT‐based body composition data. BMI was measured only once preoperatively, failing to account for dynamic weight changes associated with disease progression or treatment. Although we adjusted for several confounders, residual confounding remains possible. Smoking status and prior pelvic radiotherapy were not systematically captured in this dataset and therefore could not be included as a confounder of perioperative outcomes. Finally, as a single‐centre study, our findings may not be generalisable. Larger, multicentre studies with robust body composition data and longer follow‐up are needed to clarify the complex relationship between BMI, perioperative outcomes and survival after RC.

## 6 CONCLUSION

In this single‐centre retrospective Australian cohort, overweight BMI was independently associated with improved overall survival following radical cystectomy for bladder cancer, consistent with the obesity paradox described in the oncological literature. These findings should be interpreted with caution given the inherent limitations of retrospective single‐centre studies and may not be fully generalisable to all practice settings. However, emerging evidence suggests that this survival advantage may be time‐limited, underscoring the importance of longer term follow‐up. Additionally, obesity remains associated with increased perioperative morbidity and longer hospitalisation. These findings highlight the need for precise preoperative risk assessment and potential interventions such as prehabilitation to optimise outcomes. Further studies are required to explore tumour biology across BMI groups to determine whether the observed survival differences reflect biological variation or methodological artefacts. Additionally, future research incorporating body composition analysis and focused on the Australian health system context will be essential to guide practice and resource planning for this high‐risk surgical population.

## AUTHOR CONTRIBUTIONS

**Samiha Arulshankar:** Data curation; methodology; formal analysis; writing—original draft. **Xinyi Wei:** Data curation. **Charis Ting:** Data curation. **Tran Ngoc An Huynh:** Conceptualization; data curation; writing—review and editing. **Yashvrdhan Khanna:** Supervision; methodology; formal analysis; writing—review and editing. **Kylie Lim:** Data curation. **Munad Khan:** Supervision; writing—review and editing. **James Huang:** Supervision; writing—review and editing. **Nieroshan Rajarubendra:** Supervision; writing—review and editing. **Kevin Chu:** Supervision; writing—review and editing. **Matthew Harper:** Supervision; writing—review and editing. **Scott Donnellan:** Supervision; writing—review and editing. **Weranja Ranasinghe:** Conceptualization; supervision; writing—review and editing.

## CONFLICT OF INTEREST STATEMENT

The authors declare no conflicts of interest.
